# Long-Term Followup of Dermal Substitution with Acellular Dermal Implant in Burns and Postburn Scar Corrections

**DOI:** 10.1155/2010/210150

**Published:** 2010-12-30

**Authors:** I. Juhasz, B. Kiss, L. Lukacs, I. Erdei, Z. Peter, E. Remenyik

**Affiliations:** ^1^Department of Dermatology, Burn and Dermatosurgery Unit, University of Debrecen Medical and Health Science Center, 4032 Debrecen, Hungary; ^2^Faculty of Dentistry, University of Debrecen Medical and Health Science Center, 4032 Debrecen, Hungary; ^3^Department of Anesthesiology and Intensive Care, University of Debrecen Medical and Health Science Center, 4032 Debrecen, Hungary

## Abstract

Full-thickness burn and other types of deep skin loss will result in scar formation. For at least partial replacement of the lost dermal layer, there are several options to use biotechnologically derived extracellular matrix components or tissue scaffolds of cadaver skin origin. In a survey, we have collected data on 18 pts who have previously received acellular dermal implant Alloderm. The age of these patients at the injury varied between 16 months and 84 years. The average area of the implants was 185 cm^2^. Among those, 15 implant sites of 14 patients were assessed at an average of 50 months after surgery. The scar function was assessed by using the modified Vancouver Scar Scale. We have found that the overall scar quality and function was significantly better over the implanted areas than over the surrounding skin. Also these areas received a better score for scar height and pliability. Our findings suggest that acellular dermal implants are especially useful tools in the treatment of full-thickness burns as well as postburn scar contractures.

## 1. Introduction

Once the dermis of the injured skin is lost due to full-thickness burn, it will either be replaced by scar that originates from granulation tissue and epithelized from the wound edges, or skin grafting will close the wound. Neither the scar tissue, nor the patchy dermal residual islands of dermal papillae provided by the split-thickness skin graft (STSG) will result in a continuous healthy dermal layer to the wound site. The quality and function of dermal connective tissue and the amount and orientation of dermal elastic fibers have great impact on postinjury skin quality. The typical scar tissue is characterized by sparse elastic fibers and newly formed collagen bundles in random orientation. There are several methods available that are designed to improve the qualities of the dermal wound bed, while some of these methods aim to provide dermal tissue for replacement. Harvesting thicker grafts will enormously increase donor site morbidity; full-thickness skin grafting has a strict limitation of a mere few square cms. Donor site is often limited and when applying the gold standard of wound closure, autologous stsg, it has to be expanded to a ratio where disfiguring scarring is inevitable. A widely used alternative is to restore barrier function until definitive closure by applying temporary coverage, preferably with biological dressings. A large variety of methods are available ranging from xenograft (frog's membrane to porcine skin) to allogenic tissue (e.g., placenta, frozen-, cryopreserved- or glycerol preserved human skin). Risk of transmission of infectious diseases is a theoretical drawback of these materials; despite implementing tissue banking guidelines, this risk is relative low. There is a range of bioengineered skin substitutes available for the purpose of improving the quality of skin in the area of dermal loss. These can either be applied during tissue repair or after scar formation, as tools for reconstructive surgery. 

Due to the diversity of the commercially available biotechnologically produced materials, it is rather difficult to compare their real value and usefulness, and there remains some uncertainty regarding their effectiveness. Some of these materials contain cells, some only cellular products, characteristically in a rather complex manner. Clinically, as it seems, the simple delivery of macromolecules, such as collagen, in an unstructured manner (powder, cream, gel, etc.) may not fulfill the many requirements of the proliferative phase of wound healing. It is likely, that a 3-dimensional structure is mandatory, effectively provided by an acellular natural dermal scaffold or a tissue-engineered newly formed biological matrix. There remains a question whether the more complex cellular products could deliver the extra clinical advantage, that justifies the higher cost associated with their production. Besides the high price, challenges in obtaining reimbursement, together with complicated regulations and approval regarding the use of allogeneic cells make the future of these products uncertain [1]. The name and origin of some of the most typical and important tissue-engineered materials are summarized in Table 1 in the order of their complexity and appearance on the market. 

## 2. Acellular Preparations

MB-Collagen or Collagen-Klee (Medical Biomaterial Products GmbH Neustadt-Glewe, BRD), a resorbable, 5 mm thick collagen membrane made of porcine origin is an example of the many collagen delivery devices on the market to promote connective tissue proliferation during wound repair [2]. 

BGC Matrix (Brennen Medical Inc, St. Paul MN, USA) consists of beta-Glucan, a complex carbohydrate isolated from the cell wall of oats and a porcine collagen hydrocolloid matrix combined in a multifilament mesh [3]. 

Promogran (Systagenix Wound Management Ltd., Gargrave, UK), in addition to its bovine collagen content, also contains regenerated oxidized cellulose as an additional source of substrate for binding to matrix metalloproteases present in the chronic wounds. Substrate substitution aims at the inactivation of ECM degrading enzymes and the protection of naturally occurring growth factors. It is concluded that protease-modulation by substrate substitution acts synergistically with autologous growth factors in diabetic foot ulcers [4]. 

Biostep/Biostep Ag Collagen Matrix Dressing (Smith & Nephew Inc. St. Petersburg, FL USA) provides its porcine collagen content considerable antimicrobial activity with the combination of Silver ions (company information). 

Matriderm (Dr. Suwelack Skin & Health Care AG, Billerbeck, BRD) is a three-dimensional matrix consisting of collagen and elastin, comparable to the structure of human dermis. The sheets are used in a one-step procedure, applied dry, possess unique hemostyptical characteristics, and rehydrate “in loco” after grafting. When full-thickness acute burn wounds are treated with STSG alone, and with the use of Matriderm, the use of the device results in improved scar (skin) formation [5]. 

Alloderm regenerative tissue matrix (LifeCell Co., The Woodlands, TX USA) is a freeze-dried extracellular matrix (ECM) tissue derived from cadaver skin, through a process resulting in removal of all cellular components and residues. The ECM has high hyaluronan and proteoglycan content, while the scaffold retains its collagen and elastic fiber content and structure, with an intact basement membrane. The material is made from tissue bank skin and has a 2-year shelf life. The acellular dermal implant Alloderm is available also in a meshed version and has been advocated and approved since 1995 for the management of acute burns in which the dermis is severely damaged [6]. It allows the utilization of ultra thin autograft skin, (0,1-0,2 mm instead of 0,35–0,5 mm), strongly reducing donor site morbidity and soon allowing for reharvesting the donor area. The product in fact has received its FDA approval, as “banked human tissue” and not as a “medical device” if used in an orthotopic way, that is, as skin replacement. Probably it is the most widely used product for dermal regeneration as of now. 

The European version of Alloderm is called Glyaderm acellular dermal collagen-elastin matrix. It is a dermal substitute produced by the Euro Skin Bank (Beverwijk, NL) and is based on the glycerol-preservation technique, developed for producing allogenic temporary skin grafts for burn treatment [7]. 

SureDerm (Hans Biomed Corp., Seoul, Korea) is another similar product to Alloderm. It is made according to AATB (American Association of Tissue Banks) guidelines in South Korea, requiring less rehydration time (10 minutes versus 20–40 minutes). Only limited scientific information is available [8], but this relatively newcomer product has both FDA and CE approval, allowing to gain its share of the market. 

Integra (Integra LifeSciences Co., Plainsboro, NJ USA) dermal regeneration template consists of two layers; a porous chondroitin 6 sulphate glucosaminoglycane (GAG) and bovine collagen-based dermal analogue, which integrates with the patient's own cells and a supporting epidermal silicone sheet. The product besides promoting and regulating dermal development also provides a massive temporary cover of the wound area; the protective layer, later as the wound heals, gets peeled off. A very thin autograft is then grafted onto the neo-dermis. Integra is primarily indicated for the postexcisional treatment of full-thickness or deep partial-thickness burns and similar tissue defects. A relatively high incidence rate of infectious complications follows its application, but its frequency can be reduced by more careful timing, that is, earlier application [9].

## 3. Cell-Containing Devices

TransCyte, formerly Dermagraft TC (Advanced BioHealing Inc, Westport, CT USA) is a temporary, biosynthetic covering material composed of a semipermeable silicone membrane and human fibroblast cells of newborn foreskin origin cultured on a porcine collagen coated nylon mesh. During the 17 days manufacturing cycle in a so-called “bioreactor” the cellular products, fibronectin, tenascin, glycosaminoglycans, growth factors reach high concentration. The device is then frozen and stored at −70°C until it is used. Human application is possible, because the fibroblasts reaching the wound surface are metabolically already inactive and do not provoke immunological rejection [10]. It has been indicated for use as a temporary covering for excised burns prior to autografting. Another indication is to use it on donor sites or burns that do not require autografting, that is, as a biological dressing material [11]. 

Dermagraft (Advanced BioHealing Inc, Westport, CT USA) is similar to the previous device, but the scaffold is a biodegradable polyglactin mesh seeded with allogeneic neonatal fibroblasts. The fibroblasts proliferate and produce dermal collagen, growth factors, glycosaminoglycans (GAGs), and fibronectin to restore the dermal bed, while the mesh material is gradually absorbed. It is intended to be used as a temporary covering to promote wound healing via multiple applications of the device. The result of this type of approach is second intention healing, one reason why Dermagraft is primarily intended for diabetic foot ulcer [12]. 

Apligraf formerly Graftskin (Organogenesis Inc., Canton, MA, USA and Novartis AG Basel, CH) is a bilayered living skin equivalent composed of allogeneic fibroblasts and type I bovine collagen as dermis and allogeneic neonatal foreskin-derived keratinocytes as epidermis. It has a short shelf-life of 5 days, so needs to be applied while “fresh”. It is on the market since 1998 and is currently approved for accelerating the closure of chronic wounds, diabetic foot, and venous leg ulcer. Experimental application in excised deep burn wounds combined with autografting was reported by Waymack et al. [13]. Apligraf's DNA persisted in a minority of patients only at 4 weeks after grafting in acute partial-thickness wounds [14]. Its success in speeding the healing of acute wounds appears to be related to other factors than the persistence of donor DNA or basement membrane restoration. For the treatment of deep chronic wounds, the same manufacturer developed a monolayer product, with live human fibroblasts in a collagen matrix, without a keratinocyte layer [15], which is currently not available. A further developed version of Apligraf aiming to omit the use of xenogenic collagen and utilizing human collagen produced de novo by the human fibroblasts is in late-stage development (VCT01, company information).

OrCel (Forticell BioScience Inc., New York, NY USA) is a cryopreserved bilayered matrix with fibroblasts and keratinocytes cultured on type I. collagen 3D matrix. Its FDA approval was obtained in 2001 for treatment of acute surgical excisions, such as contracture release sites and donor sites in epidermolysis bullosa (EB) patients undergoing hand reconstruction surgery and burn surgery donor sites [16]. Due to a long period of in vitro expansion, the constiuent cells cease to express significant levels of HLA-II antigens and, upon application to a wound bed, are apparently not immediately recognized by the recipient's immune system. 

StrataGraft (Stratatech Corporation, Madison, WI USA) is a bilayered skin substitute providing a dermis and a stratified, functional epidermis generated from a pathogen-free, human keratinocyte progenitor cell line, Neonatal Immortalized KeratinocyteS (NIKS). This new device has functional performance comparable to cadaver allograft for the temporary management of burns and other complex skin defects before autografting [17]. The major advantage is its full consistency and reproducibility and a resulting additionally lower risk of disease transmission.

## 4. Long-Term Followup of Alloderm Grafts

An investigation was planned to study the beneficial effect of dermal replacement on the healing process. In authors' country, Hungary, availability of products intended to improve dermal repair is strictly limited. Not all of the previously listed materials are available. In our facility for burn treatment, we have been using Alloderm regenerative tissue matrix over 10 years for burn patients with good functional and cosmetic results. Besides Alloderm, authors have personal experience with the use of Collagen-Klee, Promogran, Integra, and Apligraf only. The indication of both Promogran and Apligraf allows only treatment of chronic wounds. Collagen-Klee and Integra were applied only on few occasions by us with promising results. The low number of cases however did not result in sufficient data allowing comparison with our regularly used product, Alloderm. In order to assess the long-term results of dermal replacement, a study was made among our burn patients who received this implant material.

## 5. Materials and Methods

We have reviewed the case documentation of 17 patients at the Burn and Dermatosurgery Unit at the Department of Dermatology, Medical and Health Science Center, University of Debrecen, Debrecen, Hungary who were grafted with a sandwich graft combining Alloderm regenerative tissue matrix and thin autologous STSG. The operations took place between the years 1996 and 2006 (Table 2). We carried out 18 operations at 17 pts whose average age was 33,7 (1,4–84) years. There were two types of operations; “early” and “late”. Early operation was regarded when the implantation of Alloderm was executed before wound closure (*n* = 10), with the intention to improve the quality of the resulting burn scar. Late operations were scar revisions (*n* = 8) done for release of contractures over a joint. The patients were operated on the average 17,8 postburn day and at the average 5,62 months postburn in the two groups, respectively. The majority of the patients had full-thickness burns, a small percentage had deep partial-thickness/full-thickness mosaic burns, the average TBSA was 35% (15%–96%). Implantation was always carried out in areas of full-thickness skin loss, either burns or excised scars. During the operations, we grafted 3140 cm^2^ s of the material, at an average of 185 cm^2^ s (6–1050 cm^2^ s). Initially we adhered to the recommendation of the manufacturer to regularly apply antibiotic solutions to the dressing, later we changed our protocol to the use of nanocrystalline silver dressings (Acticoat, Smith & Nephew Wound Management, Hull UK) as a protective overlay and administered no local antibiotics.

### 5.1. Determination of Scar Qualities

**Figure 1:**
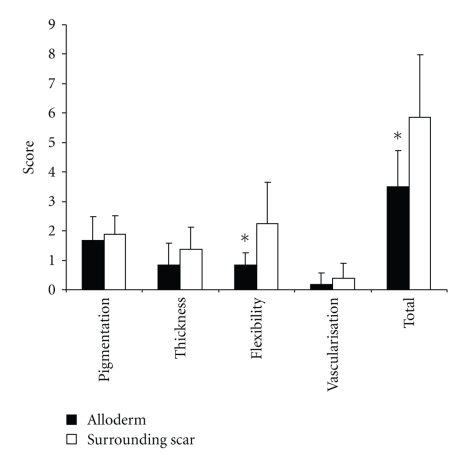
Comparison of scar qualities of the Alloderm implant sites and surrounding tissue by the modified Vancouver Burn Scar Index (VBSI).

A representative area of the dermal implant site was chosen, and its scar qualities were determined by the modified Vancouver Burn Scar Index (VBSI), described previously by Oliveira [18] upon four criteria, not including pain and pruritus. A comparable matching site of the surrounding burn scar was also chosen that received no Alloderm implant previously, and its scar qualities were also determined by the modified VBSI (Figure 1). 

### 5.2. Statistical Analysis

Results were expressed as means ± SEM.  Statistical significance between groups was determined by Student's *t*-test; *P* < .05 was considered as significant. Error bars represent ±SEM.

## 6. Results

The Alloderm implantations were extensively photo-documented, and records were made on the take-rate of the sandwich grafts, which was found to be 99%. We have seen no inflammatory reaction, no rejection, no infection, and most importantly all of the implantations resulted in soft pliable skin with acceptable cosmesis. Three of our patients died shortly (bw. 15–40 days) after the operation, their grafts were also problem free. In order to determine the long-term results of the interventions, we called the patients to control in 2007. During the control, 14 patients with 15 operation sites were investigated. The average age of scar at investigation was 50,6 months (14–122 months). Scar qualities were determined by VBSI, both of a representative area of the dermal implant site, and that of a chosen comparable matching site of the surrounding burn scar, that received no Alloderm implant previously. The VBSI measurements of the sites are collected in Table 3.

The examinations revealed uniformly better VBSI values in the Alloderm implant group than in the control group. The differences in color, thickness, and vascularization of the skin at the dermal replacement site were not significant when compared to control. Flexibility and overall scar quality however were significantly better at the Alloderm implant site than at the control area. In addition to the favorable quality of scar (skin) at the site of dermal replacement, the functional results were also excellent; all of the patients with no exception, who were operated due to scar contracture, regained their full joint movement. 

## 7. Discussion

In an elegant series of nude mouse transplantation experiments, Truong et al. [19] compared effects of dermal substitutes on wound healing. They experienced that human skin-derived products, Alloderm and their similarly processed own acellular dermal matrix produced the least contraction and the thickest neodermis in the healed wounds, when compared to control or synthetic dermal substitutes. The application of processed acellular cadaver dermis has proven extremely useful in the fields of burns and reconstructive surgery and is gaining acceptance elsewhere, for various off-label indications for example, in neurosurgery [20], ophthalmic surgery [21], head and neck surgery [22, 23], and in hernia repair [24]. Its application in extensive burns provides the grafted area with functionally and esthetically outstanding skin as a result. Despite being an additional layer between the wound bed and epithelial cover, it does not hinder graft take. Possibly due to its intact ECM structure, the repopulation by recipient cells, fibroblasts, endothelial cells, and so forth, is extremely rapid. Clinically the STSG overlay acts no differently than a simple skin graft, their color and other vitality signs are identical. There is the added advantage of the possibility of harvesting ultra thin (0,1-0,2 mm thick) skin for autografting, which dramatically reduces the trauma of the donor area. The resulting neodermis is soft, pliable resembling normal skin. In two early cases the Alloderm containing sandwich graft was applied to the decorticated bone of the scapula (unpublished case) and of the proximal third of the tibia [25]. In both cases, a vital neodermis was the result, that could be easily wrinkled and provided protective support to the overlaying epidermis. Histological examination revealed an inflammation-free neodermis, with ample vascularization and abundant loose collagen fibrils plus oligocellular elastic connective tissue. The epidermis had normal structure and keratinisation, with apparent cell proliferation (indicated by Ki67 mAb expression). The Alloderm implant converted into a compact, functionally and morphologically live neodermis as an interconnection between the epidermal layer of the skin and the underlying bony tissue [25]. In the opinion of the authors, the easy, one-step procedure of Alloderm grafting with a simultaneous thin STSG overlay is an ideal, minimally invasive procedure in case of joint contractures, delivering in most cases superior or comparable results to complicated skin or fasciocutaneous flaps without the associated donor site morbidity. 

Hereby, we present cases in which we have used this implant during the surgery of acute burns as well as in the treatment of postburn scar contractures. In our survey, we have collected data on our patients with full-thickness burns and scar contractures and assessed scar qualities compared to that of their neighboring similarly treated but nonimplanted burn scars. A total of 15 dermal replacement sites and 15 control sites were evaluated and compared. The applied VBSI results confirmed our hypothesis, namely, the implantation of acellular dermal implant makes a considerable impact on the quality of the resulting scar, by making it softer and more elastic than the surrounding tissue receiving no dermal implant. 

**Figure 2:**
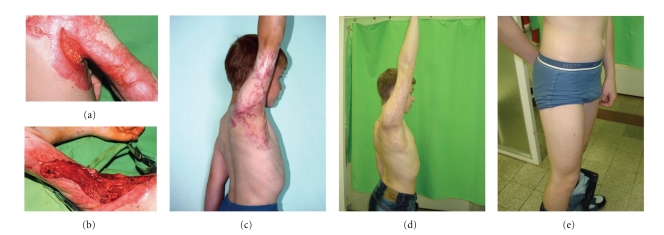
Patient no. 3, 8-years-old male suffered 4 months earlier from full-thickness burn on 20% body surface area and had conservative treatment (at another institution). (a) Upon admission: spontaneous rupture of scar that was causing severe axillary contracture. (b) Intraoperative photograph after removal of scar tissue. Full axillary function following sandwich grafting with Alloderm and thin autologous skin at (c) 3 months and (d) 7 years postoperatively. (e) No visible trace of previous harvesting of autograft skin can be seen at the donor site on right thigh (picture taken at 7 years post-op.).

A major advantage of the technique is the reduction of donor-site morbidity (Figure 2) due to the application of very thin autologous skin required.

## Figures and Tables

**Table 1:** Biotechnologically derived products designed to improve dermal wound repair.

Biotechnological device				
Name	Composition	Manufacturer	Cellular content	Ref. no
MB-Collagen/Collagen-Klee	Porcine collagen	Medical Biomaterial Products GmbH Neustadt-Glewe, BRD	no	[2]
BGC Matrix	Porcine collagen and beta-Glucan	Brennen Medical Inc, St. Paul MN, USA	no	[3]
Promogran	Bovine collagen, regenerated oxidized cellulose	Systagenix Wound Management Ltd., Gargrave, UK	no	[4]
Biostep/Biostep Ag Collagen Matrix Dressing	Porcine collagen	Smith & Nephew Inc. St. Petersburg, FL USA	no	—
Matriderm	Bovine collagen and elastin matrix	Dr. Suwelack Skin & Health Care AG, Billerbeck, BRD	no	[5]
Alloderm regenerative tissue matrix	Freeze-dried ECM from cadaver skin	LifeCell Co., The Woodlands, TX USA	no	[6]
Glyaderm dermal replacement material	Dermal collagen-elastin matrix	Euro Skin Bank, Beverwijk, NL	no	[7]
SureDerm	Freeze-dried ECM from cadaver skin	Hans Biomed Corp., Seoul, Korea	no	[8]
Integra dermal regeneration template	Bovine collagen and GAG, with a supporting epidermal silicone sheet	Integra LifeSciences Co., Plainsboro, NJ USA	no	[9]
TransCyte/Dermagraft TC temporary, biosynthetic wound cover	Porcine collagen coated nylon mesh with human fibroblast and silicone membrane	Advanced BioHealing Inc, Westport, CT USA	yes	[11]
Dermagraft	Biodegradable polyglactin mesh with human fibroblast	Advanced BioHealing Inc, Westport, CT USA	yes	[12]
Apligraf/Graftskin living skin equivalent	Bilayered structure bovine collagen with human fibroblasts and human keratinocytes	Organogenesis Inc., Canton, MA USA and Novartis AG Basel, CH	yes	[13]
OrCel cryopreserved bilayered matrix	Bilayered structure bovine collagen with human fibroblasts and human keratinocytes	Forticell BioScience Inc., New York, NY USA	yes	[16]
StrataGraft Pathogen-free human temporary allograft	Bilayered structure: non-bovine collagen with human fibroblasts and immortalized human keratinocytes	Stratatech Corporation, Madison, WI USA	yes	[17]

**Table 2:** List of patients grafted with a sandwich graft combining Alloderm regenerative tissue matrix and thin autologous split thickness skin graft.

	Age of pt. at burn	Dermal replacement			Depth of burn	TBSA %	Burn mechanism	Age of scar at control (mo)	Graft area (cm^2^)
		Type	Days after burn	Months after burn					
(1)	6	early	30		(III) degree	96	flame	exit	32
(2)	35	late		2	(III) degree, scar	25	scald	48	180
(3)	8	late		4	(III) degree, scar	20	contact	86	104
(4)	40	late		6	(III) degree, scar	15	scald	46	110
(5)	42	early	14		(III) degree	20	contact	14	200
(6)	42	early	3		(IIb-III) degree	25	contact	36	44
(7)	14	late		3	(III) degree, scar	65	electr + contact	85	130
(8)	28	early	4		(III) degree	18	contact	19	720
(9)	16	early	10		(III) degree	75	electr + contact	122	850
(10)		late		4	(III) degree, scar		electr + contact	118	200
(11)	1,8	late		22	(III) degree, scar	20	scald	34	30
(12)	1,4	early	16		(IIb-III) degree	15	scald	70	6
(13)	43	late		2	(III) degree, scar	19	scald	13	90
(14)	84	late		2	(III) degree, scar	35	flame	exit	40
(15)	47	early	27		(III) degree	55	flame	exit	110
(16)	49	early	25		(III) degree	25	contact	18	220
(17)	72	early	28		(III) degree	32	flame	36	45
(18)	43	early	21		(III) degree	39	contact	14	30
									3140

Avg.	33.66		17,8	5,62		35		50,6	185

**Table 3:** Determination of scar qualities of the Alloderm implant sites and surrounding tissue by the modified Vancouver Burn Scar Index (VBSI).

	PIGM	HEIGHT	FLX	VASC	SUM	PIGM	HEIGHT	FLX	VASC	SUM
(1)	1	1	1	0	**3**	1	2	1	0	**4**
(2)	1	1	1	0	**3**	1	2	1	0	**4**
(3)	2	0	1	0	**3**	2	2	3	0	**7**
(4)	2	0	0	0	**2**	2	1	1	0	**4**
(5)	1	1	1	0	**3**	2	1	4	0	**7**
(6)	1	0	0	0	**1**	2	1	1	0	**4**
(7)	0	0	1	0	**1**	2	2	4	0	**8**
(8)	2	1	1	0	**4**	2	2	4	2	**10**
(9)	1	0	0	0	**1**	2	1	1	0	**4**
(10)	3	1	1	0	**5**	3	2	3	0	**8**
(11)	1	2	1	1	**5**	1	1	1	1	**4**
(12)	1	0	0	0	**1**	1	1	1	0	**3**
(13)	1	0	0	0	**1**	1	1	1	0	**3**
(14)	2	0	1	1	**4**	3	0	2	1	**6**
(15)	2	0	1	1	**4**	3	0	2	1	**6**

Dermal replacement area						Surrounding scarred skin				

Abbreviations: PIGM: pigmentation, FLX: flexibility, VASC: vascularization, SUM: summary.

## References

[1] Ehrenreich M, Ruszczak Z (2006). Update on dermal substitutes. *Acta Dermatovenerologica Croatica*.

[2] Neumann-Scholz A, Lessee M, Winkler B (1988). Weiterentwicklung zum Kollagen- Hämostyptikum-Vlies. *Medicamentum Berlin*.

[3] Delatte SJ, Evans J, Hebra A, Adamson W, Othersen HB, Tagge EP (2001). Effectiveness of beta-glucan collagen for treatment of partial-thickness burns in children. *Journal of Pediatric Surgery*.

[4] Kakagia DD, Kazakos KJ, Xarchas KC (2007). Synergistic action of protease-modulating matrix and autologous growth factors in healing of diabetic foot ulcers. A prospective randomized trial. *Journal of Diabetes and its Complications*.

[5] Ryssel H, Gazyakan E, Germann G, Öhlbauer M (2008). The use of MatriDerm in early excision and simultaneous autologous skin grafting in burns-A pilot study. *Burns*.

[6] Yim H, Cho YS, Seo CH (2010). The use of AlloDerm on major burn patients: AlloDerm prevents post-burn joint contracture. *Burns*.

[7] Richters CD, Pirayesh A, Hoeksema H (2008). Development of a dermal matrix from glycerol preserved allogeneic skin. *Cell and Tissue Banking*.

[8] Lee KC, Lee NOH, Ban JH, Jin SM (2008). Surgical treatment using an allograft dermal matrix for nasal septal perforation. *Yonsei Medical Journal*.

[9] Bargues L, Boyer S, Leclerc T, Duhamel P, Bey E (2009). Incidence and microbiology of infectious complications with the use of artificial skin Integra in burns. *Annales de Chirurgie Plastique et Esthetique*.

[10] Sher SE, Hull BE, Rosen S (1983). Acceptance of allogeneic fibroblasts in skin equivalent transplants. *Transplantation*.

[11] Kumar RJ, Kimble RM, Boots R, Pegg SP (2004). Treatment of partial-thickness burns: a prospective, randomized trial using transcyte^*™*^. *ANZ Journal of Surgery*.

[12] Marston WA (2004). Dermagraft®, a bioengineered human dermal equivalent for the treatment of chronic nonhealing diabetic foot ulcer. *Expert Review of Medical Devices*.

[13] Waymack P, Duff RG, Sabolinski M (2000). The effect of a tissue engineered bilayered living skin analog, over meshed split-thickness autografts on the healing of excised burn wounds. *Burns*.

[14] Hu S, Kirsner RS, Falanga V, Phillips T, Eaglstein WH (2006). Evaluation of Apligraf® persistence and basement membrane restoration in donor site wounds: a pilot study. *Wound Repair and Regeneration*.

[15] Límová M (2002). New therapeutic options for chronic wounds. *Dermatologic Clinics*.

[16] Still J, Glat P, Silverstein P, Griswold J, Mozingo D (2003). The use of a collagen sponge/living cell composite material to treat donor sites in burn patients. *Burns*.

[17] Schurr MJ, Foster KN, Centanni JM (2009). Phase I/II clinical evaluation of StrataGraft: a consistent, pathogen-free human skin substitute. *The Journal of Trauma*.

[18] Oliveira GV, Chinkes D, Mitchell C, Oliveras G, Hawkins HK, Herndon DN (2005). Objective assessment of burn scar vascularity, erythema, pliability, thickness, and planimetry. *Dermatologic Surgery*.

[19] Truong A-TN, Kowal-Vern A, Latenser BA, Wiley DE, Walter RJ (2005). Comparison of dermal substitutes in wound healing utilizing a nude mouse model. *Journal of Burns and Wounds*.

[20] Warren WL, Medary MB, Dureza CD (2000). Dural repair using acellular human dermis: experience with 200 cases: technique assessment. *Neurosurgery*.

[21] Rubin PAD, Fay AM, Remulla HD, Maus M (1999). Ophthalmic plastic applications of acellular dermal allografts. *Ophthalmology*.

[22] Kridel RWH, Foda H, Lunde KC (1998). Septal perforation repair with acellular human dermal allograft. *Archives of Otolaryngology. Head and Neck Surgery*.

[23] Fayad JN, Baino T, Parisier SC (2003). Preliminary results with the use of AlloDerm in chronic otitis media. *Laryngoscope*.

[24] Buinewicz B, Rosen B (2004). Acellular cadaveric dermis (AlloDerm): a new alternative for abdominal hernia repair. *Annals of Plastic Surgery*.

[25] Gáspár K, Erdei I, Péter Z, Dezsö B, Hunyadi J, Juhász I (2006). Role of acellular dermal matrix allograft in minimal invasive coverage of deep burn wound with bone exposed—case report and histological evaluation. *International Wound Journal*.

